# S146. EFFECT OF CLOZAPINE ON REGIONAL CEREBRAL BLOOD FLOW IN TREATMENT-RESISTANT SCHIZOPHRENIA

**DOI:** 10.1093/schbul/sby018.933

**Published:** 2018-04-01

**Authors:** Kyra-Verena Sendt, Grant McQueen, Amy Gillespie, James MacCabe, Fernando Zelaya, Alice Egerton

**Affiliations:** Institute of Psychiatry, Psychology & Neuroscience, King’s College London

## Abstract

**Background:**

Approximately one-third of schizophrenia patients will not respond adequately to conventional antipsychotic treatment; termed treatment-resistant schizophrenia (TRS). The only antipsychotic recommended for this group is clozapine, which may have unique efficacy in improving residual symptoms. The biological mechanisms underlying its efficacy are poorly understood. Previous studies have examined the effects of clozapine on regional cerebral blood flow (rCBF) using radiotracer approaches in relatively small samples of patients, showing, in particular, frontal and limbic perfusion changes1,2,3. In this study, we evaluate the effects of clozapine on rCBF, measured with a non-invasive MRI technique - pulsed continuous arterial spin labelling (pCASL) - which does not require radiotracer injection, as part of an ongoing study to identify neuroimaging predictors and mediators of clozapine response.

**Methods:**

Participants ≥18 years of age with TRS were recruited at the Institute of Psychiatry, Psychology & Neuroscience, Kings College London (UK). TRS status was ascertained by the documented failure to respond to at least two different antipsychotic trials of adequate length. Participants were either clozapine-naïve or had not taken clozapine for at least three months prior to the baseline MRI scan. After baseline MRI, clozapine was administered as part of routine clinical care for 12 weeks, after which a second MRI scan was performed. Symptomatic response was defined as a reduction of 20% of the Positive and Negative Syndrome Scale (PANSS)4 score and non-response was defined as <20% decrease in PANSS score.

pCASL data was acquired on a General Electric 3 Tesla MR-750 MR scanner. Arterial blood was labelled using a long, adiabatic (1.8 seconds) radio frequency pulse. After a post-labelling delay of 2.025s, perfusion images were acquired with a 3D Fast Spin Echo spiral multi-shot readout (TE 32ms/TR = 5500ms; ETL = 64). Cerebral blood flow (CBF) maps were computed with a spatial resolution of 2x2x3mm, in a total acquisition time of less than 6min. CBF maps were pre-processed using the Automatic Software for ASL processing (ASAP) toolbox5. Changes in rCBF after 12 weeks of clozapine were analysed in a full factorial ANOVA design, using SPM 12 (www.fil.ion.ucl.ac.uk/spm). Clusters of significant CBF changes were assessed at p<0.05 after Family-Wise Error correction for cluster extent, using a cluster-forming threshold of T>2.74.

**Results:**

This is an interim analysis of 24 patients who completed both scans. Contrasts were examined at a whole brain, assumption-free voxel-wise analysis, restricted to grey matter and co-varied for global perfusion. Clozapine administration significantly decreased perfusion in the medial frontal gyrus. There was also a significant response x time interaction, centred in the left posterior cerebellum and extending to the bilateral visual cortex and right precuneus.

**Discussion:**

These interim results indicate that pCASL may be able to identify brain regions in which activity is modulated by clozapine administration as well as areas that may mediate symptomatic improvement. A key question for future analyses will be the degree to which rCBF may predict symptomatic response to clozapine, as the ability to predict a good likelihood of response could enable earlier clozapine initiation.

**References:**

1. Lahti AC et al. Biol Psychiatry 2003; 53: 601–8.

2. Potkin SG et al. Mol Psychiatry 2003; 8: 109–13.

3. Molina V et al. Prog Neuropsychopharmacol Biol Psychiatry 2008; 32: 948–54.

4. Kay SR et al. Psychiatry Res 1988; 23: 99–110.

5. Mato Abad VM et al. Magn Reson Imaging 2016; 34: 334–44.

